# Can self-reported disability assessment behaviour of insurance physicians be explained? Applying the ASE model

**DOI:** 10.1186/1471-2458-11-576

**Published:** 2011-07-19

**Authors:** Antonius JM Schellart, Romy Steenbeek, Henny PG Mulders, Johannes R Anema, Herman Kroneman, Jan JM Besseling

**Affiliations:** 1 VU University Medical Center, Department of Public and Occupational Health, EMGO Institute for Health and Care Research, Amsterdam, the Netherlands; 2 TNO Work and Employment, PO Box 718, 2130 AS Hoofddorp, the Netherlands; 3 UWV, Employee Benefits Insurance Authority, Amsterdam, the Netherlands; 4 Research Center for Insurance Medicine, AMC-UWV-VUmc, the Netherlands

## Abstract

**Background:**

Very little is known about the attitudes and views that might underlie and explain the variation in occupational disability assessment behaviour between insurance physicians. In an earlier study we presented an adjusted ASE model (Attitude, Social norm, Self-efficacy) to identify the determinants of the disability assessment behaviour among insurance physicians. The research question of this study is how Attitude, Social norm, Self-efficacy and Intention shape the behaviour that insurance physicians themselves report with regard to the process (Behaviour: process) and content of the assessment (Behaviour: assessment) while taking account of Knowledge and Barriers.

**Methods:**

This study was based on 231 questionnaires filled in by insurance physicians, resulting into 48 scales and dimension scores. The number of variables was reduced by a separate estimation of each of the theoretical ASE constructs as a latent variable in a measurement model. The saved factor scores of these latent variables were treated as observed variables when we estimated a path model with Lisrel to confirm the ASE model. We estimated latent ASE constructs for most of the assigned scales and dimensions. All could be described and interpreted. We used these constructs to build a path model that showed a good fit.

**Results:**

Contrary to our initial expectations, we did not find direct effects for Attitude on Intention and for Intention on self reported assessment behaviour in the model. This may well have been due to the operationalization of the concept of 'Intention'. We did, however, find that Attitude had a positive direct effect on Behaviour: process and Behaviour: Assessment and that Intention had a negative direct effect on Behaviour: process.

**Conclusion:**

A path model pointed to the existence of relationships between Attitude on the one hand and self-reported behaviour by insurance physicians with regard to process and content of occupational disability assessments on the other hand. In addition, Intention was only related to the self reported behaviour with regard to the process of occupational disability assessments. These findings provide some evidence of the relevance of the ASE model in this setting. Further research is needed to determine whether the ASE variables measured for insurance physicians are related to the real practice outcomes of occupational disability assessments.

## Background

It is common knowledge that doctors tend to vary in their patient assessments and thus may draw different conclusions for the diagnosis and treatment of comparable cases [1-7]. Targeted research has shown that General Practitioners (GPs) are no exception [8-16]. When it comes to occupational disability, doctors are required to perform very specific tasks. In some countries sick-leave certificates are issued by the GP whereas in others, including the Netherlands, occupational or insurance physicians asses incapacity for work. Issuing sick-leave certificates can prove a complicated process for GPs, especially when faced with chronic, complex or dubious cases [17]. Variations were found in both the frequency [18-20] and duration [21,22] of sick-leave certificates signed by GPs. Variations were also found in the outcomes of long-term incapacity assessments by insurance physicians. Ydreborg and Ekberg [23] found variations in the rejection of applications for disability pension. Spanjer et al. [24] found that, on the basis of written reports, the inter-rater reliability of physical and mental disability assessments by insurance physicians was reasonable to good. It was, however, poor for assessments of the number of hours that patients could function daily. They also found a significant difference in the scores that insurance physicians accorded for work limitation [25].

In this paper we examine assessments of work limitations of employees on long-term sick-leave by insurance physicians in the Netherlands. In the Netherlands, if you are partially or fully incapable of working after two years of illness, you may be eligible to receive a benefit under the Work and Income Act (WIA). The WIA succeeded the Disability Insurance Act (WAO) in January 2006. The Adapted Re-assessment Act (HERBO) was introduced in August 2004 for the reassessment of WAO benefits clients, i.e. the claimants (< 50 years), on the basis of new, stricter criteria that put the emphasis on the client's residual functional capacities. The WAO and WIA differ in the time of assessment. The WAO provides for assessments after one year of sick-leave, whereas the WIA provides for assessments after two years.

Though it is generally acknowledged that variations will occur in the assessment of comparable cases, legislation, protocols and measures were introduced to narrow down this variation and promote uniformity [26].

In order to assess a clients' work limitations the insurance physician starts with the functional limitations experienced by the client. These are tested for plausibility and internal and external consistency on the basis of the medical history and the actual ability of the client to perform tasks (Medisch Arbeidsongeschikheidscriterium, MAOC/medical criteria guidelines for occupational disability). The insurance physician bases his assessment on an interview and possibly a physical examination. He can obtain additional information by ordering additional tests or by contacting the GP, specialist or other healthcare provider, or the occupational physician (OP) who assessed the first two years of disability. The client's capacity for work is determined by reference to an instrument known as FAL (Functional Ability List). On this list the physician enters the client's scores for limitations and abilities. These findings serve as the input for the labour expert in determining the extent to which the client is able to earn income and able to work. As an instrument the FAL comes within the statutory framework of disability assessments in the Netherlands.

The outcome of work limitation assessments by insurance physicians can be seen as the result of behaviour influenced by various factors, including behavioural determinants of the physicians in relation to the intended object of their assessment. Little is known of what considerations and views of insurance physicians may partly account for variation in the outcome of assessments. Therefore, a study was launched to add to the sparse knowledge in this field. First, Steenbeek et al. [27] developed measurement instruments for assessment behaviour and its determinants, on the basis of the Attitude - Social norm - self Efficacy (ASE) model. They defined assessment behaviour as all behaviour that may influence the outcome of the assessment, including the collection and evaluation of information about the client. They explain how the ASE model [28] was based on Azjen's 'Theory of Planned Behaviour (TPB)' [29], supplemented by elements from the 'Social Cognitive Theory (SCT)' of Bandura [30]. ASE is a model that has general scientific acceptance and explains behaviour by linking Attitude, Social Norm and Self-efficacy with Behaviour and behavioural Intention [31]. In addition to the three determinants of Intention and behaviour, factors such as 'Knowledge' and 'Barriers' can play a role. TPB and the ASE model are used in the Netherlands to explain, among other things, the behaviour of physicians and patients in an occupational health context [32-36] and the health behaviour of individuals who belong to a particular target group [37,38]. On the basis of TPB research Croon & Langius [33] have studied attitudes and working styles (behavioural intentions) among insurance physicians. The present survey takes the ASE model as the basis for possible explanations of the behaviour of insurance physicians in assessing work disability after sick-leave lasting one year (Disability Insurance Act-WAO), two years (Work and Income Act - WIA) or more years (Adapted Reassessment Act - HERBO).

**Figure 1:**
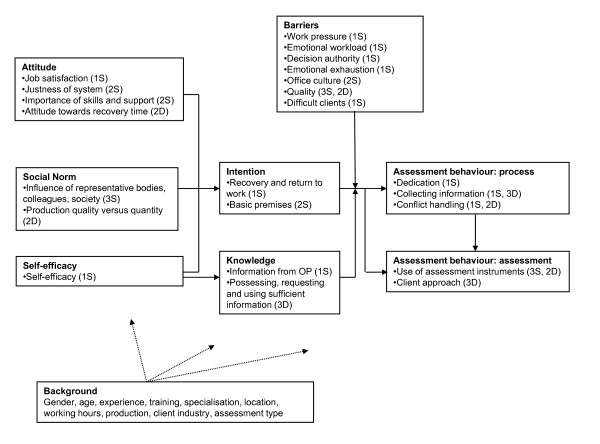
**Research model**. From Steenbeek et al. [27]. S = Scale; D = Dimension, the number refers to the number of constructed scales and dimensions (measures).

During the first part of the present study a questionnaire was developed, based on literature study and interviews. It was completed by 231 insurance physicians. These formed the basis for the development of scales and dimensions that fitted with the concepts of the ASE model (Figure 1). Hereby, the ASE model was slightly adjusted: behaviour was divided into two blocks: behaviour that reflects 1) the assessment process and 2) assessment behaviour. This modified ASE model pretends to describe the assessment behaviour of insurants physicians and the determinants of this behaviour. The aim of the present paper is to confirm the model of Figure 1. Our research questions are 1) is there a model with a good fit?, 2) how is the self-reported behaviour of insurance physicians associated with Attitude, Social norm, Self-efficacy and Intention while taking account of Knowledge and Barriers?, and 3) how can we interpret relations within the resulting model?

## Methods

### Study design and procedure

The research group of the organizations participating in this study - TNO Quality of Life, the EMGO Institute of the VU Medical Centre and the Employee Benefits Insurance Agency (UWV) - drafted the questionnaire for insurance physicians who perform disability assessments of long-term sick-listed employees ("clients"). At the start of 2008 UWV drew up a list of addresses of all insurance physicians working for the agency. In March 2008 UWV sent the questionnaire, together with a covering letter containing an invitation to participate in the research, to the home addresses of insurance physicians. A reminder was sent two weeks later. Not all the physicians belonged to our target group, but it was not possible to make a selection in the mailing. In total we wrote to 750 insurance physicians. Our estimate was that the target group consisted of 450 insurance physicians: insurance physicians actively employed by UWV in May 2008 who had performed work disability assessments of long-term sick-listed employees in 2007 or in preceding years. The criteria for inclusion were mentioned in the accompanying letter. The participants sent the completed questionnaire to TNO (Netherlands Organization for Applied Scientific Research). The response consisted of 231 questionnaires (estimated response approximately 51% of the target group).

As we lacked the necessary data of the target population to do a full non-response analysis, we checked whether the group of participants (N = 231) was representative of the total population of insurance physicians working for UWV (N = approximately 900) in terms of age, gender, and working hours per week.

As this study was based on a survey under (insurance) physicians only, approval by a Medical Ethical Commission was not necessary under Dutch law.

### Questionnaire

In drawing up the questionnaire we used existing and newly developed concepts. These concepts were chosen on the basis of literature studies and four interviews with insurance physicians. In a pilot study two insurance physicians completed the questionnaire while thinking aloud in order to enable us to test whether the questions were correctly understood. Finally, two other insurance physicians were timed while they completed the questionnaire. For a detailed description of the questionnaire, the operationalization of the constructs of the ASE-model, the construction of measurements, and the imputation of missing values we refer to Steenbeek et al. [27]. Below we give a summary of the construction and content of the measures.

### Construction of measures

The operationalization of the theoretical concepts of the ASE-model and the validation of the subsequent measurements are described in detail by Steenbeek et al. [27]. We operationalized the theoretical concepts of the ASE model with 28 additive scales and 20 dimensions. An *additive scale *is constructed of numerical categories of items that can be meaningfully added. Some additive scales were formed on basis of already validated scales; other scales were formed after extracting factors and performing reliability analysis. For some items it was not possible to construct an additive scale. We grouped these items on a theoretical basis and used homogeneity analysis to analyse the *dimensions *behind these grouped items. The object scores of the dimensions that were meaningful and gave additional information were selected as variables. We call these variables 'dimensions' (indicated by 'D' in Figure 1), contrary to the variables which we constructed as additive scales, which we call 'scales' (indicated by 'S' in Figure 1). We did not reverse individual scales and dimensions so that higher scores meant a more positive outcome.

### Content of measures

The meaning of all measurements (28 scales and 20 dimensions) is presented separately (see Additional file 1). The measures of Attitude reflect the attitude towards the insurance physician's own job [39], development of skills, support by management, the WIA and the social security system. The measures of Social norm reflect how much the insurance physician is influenced by the staff, the employer (UWV), employee representative bodies, colleagues and society in general. The Self-efficacy scale of Scholtz et al. [40] was adjusted for this study and measures the Self-efficacy specific for behaviour performed during the assessment interview, e.g. the ability to resolve difficult issues involving clients, the ability to stick to the proper procedure when a client is being difficult, the ability to stick to the planned course of the assessment interview, the ability to have a number of different solutions available when faced with a problem during the assessment interview, the ability to find a way of dealing with the situation no matter what happens during the assessment interview, etcetera. Various aspects of Barriers were measured: cooperation and co-determination [39], work pressure [41], emotional workload [42], decision-making authority [43,44], emotional exhaustion [45], experiencing difficult clients and divers stimuli for the quality of the assessment work of insurance physicians. The measures of Knowledge reflect whether insurance physicians feel they possess sufficient medical information and knowledge. The measures of Intention reflect to what extent insurance physicians intend to perform disability assessments according to the professional standards of their professional organization, the disability legislation, and of their employer, UWV. These standards regard the importance of three aspects of objectives, task-setting and criteria of insurance physicians in the Netherlands, which concern their core business: a) stimulate recovery, return to work, self-perception and reintegration, b) estimate residual capacity, sickness, disorders, limitations and handicaps, and c) collect a consistent and verified account of daily activities of the client. The measures of Behaviour with respect to the assessment process reflect conflict handling [46], dedication [47], and various aspects of the insurance physician's assessment interview technique. Finally, measures of Behaviour with respect to the assessment itself reflects compliance with permanent full disability rules, and whether or not expressing a formalistic approach towards a clients' possibilities and limitations.

### Background variables

In the questionnaire, we measured the following background variables: gender, registered as insurance physician, additional medical specialization (GP, OP, medical specialist), working hours (week), years of experience as an insurance physician, assessments mainly under WIA or WAO or Wajong (Invalidity Insurance for Young Disabled Persons Act) legislation, client's industry/sector (agriculture, fishing and food, construction and timber, manufacturing, retail and wholesale, transport, financial services, temporary work, health, education, rest of public sector, other).

### Analysis

Our original purpose was to estimate a structural equation model with latent variables for the theoretical constructs of the ASE model, using Lisrel [48]. This proved impossible, however, as the operationalized model was fairly complex with 21 background variables and 48 additive scales and dimension scores [27] (see Figure 1). Furthermore, the number of cases (n = 231) did not allow us to reduce the number of variables with a structural equation model that included latent variables. In addition, the individual scales and dimensions of an ASE-construct are not suited to form one consistent composite scale, i.e. the individual scales and dimensions cannot be regarded as items that can be meaningfully added to each other. We therefore decided to reduce the number of variables (scales and dimensions) by separately estimating each theoretical ASE construct as latent variables in measurement models, which have to be considered a part of the results of the estimated model. Self-efficacy formed an exception because it consisted of only one scale. This procedure reduced the 48 scales and dimensions to eight ASE variables: Attitude, Social norm, Self-efficacy, Barriers, Knowledge, Intention, Behaviour: process and Behaviour: assessment.

The factor loadings were calculated as standardized coefficients. Factor scores are individual scores on basis of the factor loadings and the individual scores of the observed scales and dimensions on which a latent variable loads. The factor scores were treated as observed variables when we estimated a path model: a model with only observed variables.

Before assessing the various measurement models, we normalized the scales and the dimensions by applying the normal score procedure [49], using Prelis 2.72 [50]. The scales and dimensions were assigned to an ASE construct according to Figure 1. Maximum Likelihood was used as estimation procedure. Because we did not reverse individual scales and dimensions, factor loadings of the latent ASE-variables could have positive or negative coefficients. Non-significant loadings of a latent ASE-variable on the observed variables were deleted. We chose p < 0.10 as the level of significance because we did not want to miss any interesting relationships. Associations between measurement errors of the observed variables were allowed if suggested by the Lisrel program (Modification Indices > 3.84). The model fit was good [51] if the Chi-Square of the model was less than twice the number of degrees of freedom, if the Root Mean Square Error of Approximation (RMSEA) and the Standardized Root Mean Square Residual (SRMR) were both smaller than 0.05, and if the Comparative Fit Index (CFI), was equal to or greater than 0.90. We verified that the standardized residuals were normally distributed.

The direction of the factor score of Social norm was reversed so that all factor scores of the ASE-constructs indicated a higher score on the meaning of the ASE-construct. Because it proved impossible to estimate a measurement model for the latent variable Knowledge alone (i.e. a not positive definite correlation matrix of measurement errors), we estimated the latent variable Knowledge and the latent variable Barriers in the same measurement model, because theoretically both play a role in the relationship between Intention and Behaviour in the ASE model. Furthermore, because the measurement model of Intention proved to be a Heywood case [52], i.e. a (saturated) model with a standardized factor loading of 1.05 for one of the three scales and a negative disturbance term (-0.10), we fixed the standardized measurement error of this scale at 0.05.

For the path model the background variables were defined as exogenous variables. The scale for Self-efficacy and the factor scores for the other seven ASE constructs were defined as endogenous variables. Because all variables, including the factor scores for the ASE-constructs, were treated as observed variables, we presented them as rectangles instead of ovals in the figures, according to the convention in path analysis [48]. In the start model all the exogenous variables could have a direct effect on the endogenous variables. The direct effects between the endogenous variables were specified in accordance with the theoretical ASE model in Figure 1. We began by selecting the significant (p ≤ 0.10) estimated parameters of the exogenous on the endogenous variables. Then we fitted the model by adjusting the parameters for the endogenous variables on other endogenous variables one by one, i.e. by closing non-significant parameters (p > 0.10) and opening parameters with the largest Modification Index (at least > 3.84) if this was theoretically justified. These adaptations did not include direct effects between the determinants Attitude, Social norm and Self-efficacy, because direct effects between these determinants are not justified on theoretical grounds. Instead, relationships between these three variables could be opened as associations between the disturbance terms of these endogenous variables. For the fit of the estimated path model we used the same kind of model fit parameters as for the measurement models. In addition we ensured that the correlations of the parameter estimates were < 0.7: large correlations may indicate that the model is nearly non-identified and that some of the parameters cannot be determined from the data [48]. We analysed the product moment correlation matrix, resulting in the estimation of standardized parameters. We used the Lisrel program [50] to estimate both the measurement models and the structural model.

## Results

### Descriptives

The mean age of the participants (N = 231) was about 51 years (sd = 7), about 41% were female, and they worked on average about 33 hours a week. The participants did not substantially differ from the population of insurance physicians (N = approximately 750) in this respect. About 86% was registered as insurance physician and about 15% was also registered in another medical speciality. The average number of performed assessments per week was about 9 (sd = 4). Insurance physicians had on average 16 years (sd = 8) of experience. We refer to Steenbeek et al. [27] for further details.

### Measurement models of latent variables

The seven factor score variables were normally distributed (with mean = 0 and variance = 1) as was the scale for Self-efficacy (median = 32,0; mean = 32.81; sd = 4.21). The factor loadings for all latent variables and the model fit parameters are listed in table 1. All measurement models for the latent variables showed a good fit.

**Table 1:** Results measurement models of latent variables

	Standardized factor loadings of latent variables						
	
Scale/dimension (standardized measurement error term)	A	SN	B*	K*	I^#^	BP	BA
Job satisfaction (0.84)	0,40						
Positive attitude towards WIA (0.93)	0,26						
Social security system just (0.95)	0,23						
Quality: development of skills important (0.54)	0,68						
Quality: support by management important (0.78)	0,47						
Managing by reference to quality rather than quantity, reversed (0.98)		-0,16					
Managing less by reference to production targets and outcomes, reversed (0.97)		-0,16					
Opinion of UWV and employee representative bodies important, reversed (0.35)		0,80					
Colleagues' opinion important, reversed (0.32)		0,82					
Society's opinion important, reversed (0.87)		0,37					
Work pressure (0.90)			0,31				
Emotional workload (0.93)			0,26				
Decision making authority (0.86)			-0,38				
Emotional exhaustion (0.88)			0,35				
Office culture: good cooperation (0.49)			-0,72				
Office culture: sufficient co-determination (0.39)			-0,78				
Quality: influence of staff physician beneficial (0.88)			-0,34				
Quality: influence of refresher training and consultation beneficial (0.96)			-0,19				
Quality: influence of manager beneficial (0.80)			-0,45				
Quality: influence of legislation and reorganisations not adverse (0.88)			-0,35				
Many difficult clients/cases (0.89)			0,34				
Sufficient information from the occupational physician (0.75)				0,49			
Possessing, requesting and using insufficient information (0.95)				-0,22			
Sufficient knowledge, reintegration report less often supplements medical information (0.86)				-0,37			
Stimulate recovery and return to work (0.83)					0,41		
Basic premises: residual capacity (0.93)					0,27		
Basic premises: client's account and home circumstances (0.01)					0.99		
Dedication (0.89)						0,34	
Technical interview: describe object and procedure (0.89)						0,34	
Interview management: client decisive (0.86)						-0,38	
Conflict handling: seek compromise (0.91)						0,30	
Comply with permanent full disability rules (0.82)							0,43
FAL: take account of client (0.81)							-0,43
FAL: consult with labour expert when not necessary (0.91)							-0,29
FAL and recovery time: focus on impairments (0.80)							0,45
Client approach: involved with and time for (0.84)							0,40

Fit measurement model							
Chi-square	1.47	0.29	55.9*		0.03	0.35	1.67
Degrees of freedom	3	3	66*		1	1	3
Probability	0.69	0.96	0.81*		0.85	0.56	0.64
Root Mean Square Error of Approximation	0.00	0.00	0.00*		0.00	0.00	0.00
Comparative fit index	1.00	1.00	1.00*		1.00	1.00	1.00

A = Attitude; SN = Social norm; B = Barriers, K = Knowledge; I = Intention; BP = Behaviour: process; BA = Behaviour: assessment.

* Barriers and Knowledge were estimated in one measurement model.

# Intention was estimated with the value of the measurement error of 'Basic premises: client's account and home circumstances' fixed at 0.05 to avoid a Heywood case.

#### Attitude

A higher score for the latent variable 'Attitude' implied greater job satisfaction, a positive attitude towards the Work and Income (Capacity for Work) Act and the social benefits system, and a stronger belief in the development of one's own competence and support by management. In short, the variable points at a positive attitude towards both the profession and the system. Two dimensions were omitted from the measurement model: 'Recovery time: client still has some energy left after work' and 'Recovery time: good relationship with client'.

#### Social norm

A higher score for the latent variable 'Social norm' implies that the management is more based on quantity, production or outcomes and that more importance is attached to the opinion of the Employee Insurance Benefits Agency and employee representative bodies, fellow insurance physicians and society at large. In short, a higher score represents the insurance physicians who attach more importance to the opinion of others.

#### Barriers and Knowledge

Barriers and Knowledge were included as two separate latent variables in one measurement model because of technical reasons. The resulting outcome of this measurement model is in line with our allocation in the ASE model, i.e. both latent ASE-constructs loaded on items that were theoretically assigned to these ASE-constructs.

#### Barriers

A higher score for the latent variable 'Barriers' implies a respondent who experiences more work pressure, higher levels of emotional workload, less decision-making authority, higher levels of burnout, and less cooperation and input at office level. It also indicates that certain factors are experienced as restraints of the quality of the assessments, namely: supervisory insurance physicians, refresher courses and consultation, managers, legislation and reorganizations, and difficult groups of clients. In other words, a higher score for this variable means that the insurance physician is experiencing more barriers. Only the dimension 'Quality: influence of guidelines not adverse and production target not beneficial' was omitted from the model.

#### Knowledge

A higher score for 'Knowledge' implies that the respondent gets sufficient information on the client from the OP, sufficient medical and other information from third parties and is more likely to find that the Reintegration Report supplements the medical information. All in all, the insurance physician feels that he has enough knowledge and information to perform the assessment. Only the dimension 'Insufficient medical information and knowledge' was omitted from the model.

#### Intention

A higher score for 'Intention' implies that the respondent attaches more importance to: the promotion of recovery, resumption of work, self-reflection and re-integration, the relevance of capability, illness, disorders and handicaps in the assessment and, most of all, proper checks for the consistency of information on the daily activities and home situation of the client. To some extent this paints the professional attitude that one would expect from an insurance physician. We can therefore refer to it as 'professional intention'.

#### Behaviour: process

A higher score for the latent variable 'Behaviour: process' indicates that the respondent shows higher levels of dedication, pays explicit attention to purpose and procedure during the interview, determines the order of the discussion topics and looks for a compromise in the event of a difference of opinion. A higher score for Behaviour: process therefore suggests an insurance physician who is eager, takes control and is prepared to strike a compromise with the client.

The variables 'Interview: limitations not checked', 'Interview: respond to client', 'Conflict handling: engage in confrontation' and 'Conflict handling: play down differences' were omitted from the model.

#### Behaviour: assessment

A higher score for the latent variable 'Behaviour: assessment' indicates that the respondent assigns permanent and full disability (in the Netherlands known as GBM) according to the guidelines, focuses less on the specific situation of the client (home situation and future) when drawing up the FAL (functional abilities list), seldom or never consults a labour expert unless absolutely necessary, works with precision when drawing up the FAL and is meticulous in determining disorders and limitations. This insurance physician also believes that he takes time for and is involved with the client. A higher score for 'Behaviour: assessment' is therefore typical of an insurance physician who sticks to the guidelines, does not consider the specific situation of the client, thinks as he goes along, but still believes that he engages with the client.

The variables 'FAL and recovery time: stringent/formalistic approach', 'Client approach: time for account of daily activities and reporting' and 'Client approach: too little time, but involved with' were omitted from the model.

### Model results

#### Final model: fit parameters

**Figure 2:**
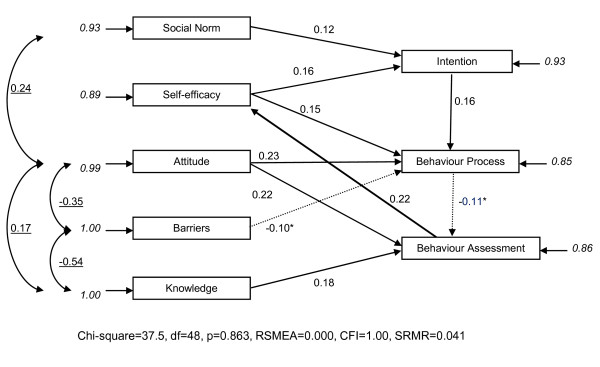
**Final Lisrel model: direct effects of endogenous variables on endogenous variables, disturbance terms and correlations between disturbance terms**. For all coefficients p ≤ 0.05; except for the marked coefficient (*) p = 0.10. Italic coefficients are disturbance terms; underscored coefficients are correlations between disturbance terms; the other coefficients (not italic and not underscored) are direct effects. All coefficients are standardized. Relations are positive unless marked with a minus sign (-). df = degrees of freedom; p = probability; RSMEA = Root Mean Square Error of Approximation; CFI = Comparative Fit Index; SRMR = Standardized Root Mean Square Residual

We modelled the direct effects between the endogenous variables as shown in Figure 1. Because this model did not fit well, we then developed, via various steps (see Additional file 2), a final model (see Figure 2) with a good fit (Chi-square = 37.5, df = 48, p = 0.863, RSMEA = 0.000, CFI = 1.00, SRMR = 0.041).

#### Final model: correlations between exogenous variables

The correlations between the exogenous variables are shown in Table 2. Only three significant correlations were found (p ≤ 0.05): between 'majority of clients from retail sector'and 'years of experience' (r = -0.15), and 'majority of clients from industrial sector' (r = 0.27), respectively, and between 'majority of clients from industrial sector' and 'disability assessments mainly under the Disability Insurance Act legislation' (r = -0.14).

**Table 2:** Final model, correlations between exogenous variables

Exogenous variables	Med spec	Hours/ week	Years exp	Industrial	Retail	WAO
Med spec	-					
Hours/week	-.0.01	-				
Years experience	-.0.09	-0.09	-			
Industrial	0.03	-0.09	0.06	-		
Retail	0.04	0.10	-0.15*	0.27**	-	
WAO	-0.12#	-0.05	.-0.05	-0.14*	-0.01	-

Med spec = additional medical specialization; Hours/week = working hours per week; Years exp = years experience as insurance physician; Industrial = majority of clients from industrial sector; Retail = majority of clients from retail sector; WAO = disability assessments mainly under the Disability Insurance Act legislation.

# = p ≤ 0.10; * = p ≤ 0.05; ** = p ≤ 0.01; other off-diagonal correlations are not significant.

#### Final model: the influence of exogenous on endogenous variables

Some exogenous variables turned out to have relationships with the ASE concepts (see Table 3). The completion of additional training in a medical specialization was associated with being more influenced by others (Social norm), higher Self-efficacy and a less stringent, guideline-based, disorder-oriented approach (Behaviour: assessment). A contract for a higher number of working hours a week was associated with being less influenced by others (Social norm), higher Self-efficacy, a less professional Intention and a more stringent, guideline-based, disorder-oriented approach (Behaviour: assessment). More years of experience as an insurance physician was associated with a less positive Attitude to the profession and the system, being less influenced by others (Social norm), higher Self-efficacy and the experience of insufficient medical information and information from third parties (Knowledge). A large percentage of clients from the industry sector was accompanied by a more stringent, guideline-based, disorder-oriented approach (Behaviour: assessment) whereas a large percentage of clients from the retail sector was accompanied by a less stringent, guideline-based, disorder-oriented approach (Behaviour: assessment). At last, performing disability assessments mainly under the Disability Insurance Act legislation (WAO) was associated with being more influenced by others (Social norm).

**Table 3:** Final model, standardized direct effects of exogenous variables on endogenous variables

	Endogenous variables							
	
Exogenous variables	Attitude	Social norm	Self-efficacy	Barriers	Knowledge	Intention	Behaviour: process	Behaviour: assessment
Med spec		0.12#	0.15*					-0.13*
Hours/week		-0.13*	0.13*			-0.19**		0.17**
Years exp	-0.14*	-0.13*	0.21**		-0.12*			
Industrial								0.17**
Retail								-0.13*
WAO		0.18**						
R2-structural equation	0.018	0.078	0.109	0.000	0.014	0.071	0.137	0.150

Med spec = additional medical specialization; Hours/week = working hours per week; Years exp = years experience as insurance physician; Industrial = majority of clients from industrial sector; Retail = majority of clients from retail sector; WAO = disability assessments mainly under the Disability Insurance Act legislation.

# = p ≤ 0.10; * = p ≤ 0.05; ** = p ≤ 0.01

#### Final model: associations between disturbance terms of endogenous variables

The disturbance term of the Attitude towards the profession and the system was positively associated with the disturbance terms of 1) being attached to the opinions of others in general (Social norm), 2) having the experience of sufficient information and knowledge (Knowledge), and negatively associated with 3) that of the experience of barriers (Barriers) (see Figure 2). The disturbance term of experiencing sufficient information and knowledge (Knowledge) was also negatively associated with that of experiencing barriers (Barriers).

#### Final model: direct effects between endogenous variables

The direct effects between endogenous variables in the final model are shown in Figure 2. Being more influenced by others (Social norm) and a higher Self-efficacy lead to a higher Intention to adopt the - expected - professional Attitude. A positive Attitude towards the profession and the system, higher Self-efficacy, low Barriers and more of the - expected - professional Intention lead to an increase in Behaviour: process, eager physicians who take control and are prepared to strike compromises.

A positive Attitude towards the profession and the system, and the experience of sufficient Knowledge were related to an increase in 'Behaviour: assessment': strict insurance physicians who stick to the rules and focus on the disorder. But more eagerness, control and compromise (Behaviour: process) was associated, although weakly, with less 'Behaviour: assessment'.

The model showed a significantly better fit when the path from Self-efficacy to Behaviour: assessment was replaced by a direct path from Behaviour: assessment (stringent, guideline-based, disorder-oriented and involved) to Self-efficacy (Chi-square = 37.5, df = 48, p = 0.863, RSMEA = 0.000, CFI = 1.00, SRMR = 0.041, versus Chi-square = 42.5, df = 48, p = 0.697, RSMEA = 0.000, CFI = 1.00, SRMR = 0.046). In plain terms, stringent and disorder-oriented physicians develop more Self-efficacy, which leads them to adopt more of the - expected - professional Intention and to perform more eager, in control and being prepared to compromise (Behaviour: process). This, in turn, has a (non-significant) negative effect on stringent, disorder-oriented assessments (Behaviour: assessment).

The explained variances were for Intention 0.07, for Behaviour:: process 0.14 and for Behaviour: assessment 0.15 (see Table 3).

## Discussion

### Findings

We estimated latent ASE constructs that represented most of the assigned scales and dimensions. All of them could be described and interpreted. They were then used to build a path model that showed a good fit.

One noteworthy aspect of the final model is the positive connection between Behaviour: assessment and Self-efficacy. An insurance physician who is stringent, guideline-based and disorder-oriented strengthens his sense of Self-efficacy. This in turn strengthens the professional Intention and leads to greater eagerness, control and willingness to compromise, after which the formalistic behaviour tends to decline (see Figure 2). This seems to indicate a striking feedback mechanism in the model.

If we look at the triangle of Attitude, Behaviour: process and Behaviour: assessment, we see that Attitude has two positive direct effects on Behaviour: process and Behaviour: assessment and we see a negative direct effect between Behaviour: process and Behaviour: assessment. This may appear contradictory but one should bear in mind that Behaviour: process and Behaviour: assessment are not opposites (the direct effect between the two is also weak) so it is perfectly feasible for the same - and other - aspects of Attitude to exert a positive effect on both.

The positive connection between a positive attitude to the profession and system (Attitude) and being more influenced by others (Social norm) seems to concur with the positive direct effect of being more influenced by others (Social norm) on greater professional intention (Intention). This indicates that insurance physicians that perform more according to the norms of others have a stronger professional Intention and, at the same time, feel more positive about the profession and the social benefits system.

Another notable finding is the direct negative effect of experience of more barriers (Barriers) on eagerness, taking control and willingness to compromise (Behaviour: process) as opposed to the direct positive effect of receiving knowledge and information more frequently (Knowledge) and sticking to the rules (Behaviour: assessment). It is conceivable that physicians who stick more to the rules have a greater need for knowledge and information (about guidelines, third parties etc.). The latter profile goes hand in hand with a positive attitude to the profession and the social benefits system (Attitude). On the other hand, insurance physicians who experience more barriers (Barriers) - expressed as heavier work pressure and more frequent tiredness - may be less eager and feel more often that they have no control over the course of the assessment interview (Behaviour: process). The experience of more barriers also goes hand in hand with a less positive attitude to the profession and the social benefits system.

In contrast to what was hypothetically expected of the ASE model, we found no direct effect for Attitude on Intention. This may be related to the fact that only three aspects were incorporated in Intention: promote recovery; importance attached to capability in the assessment; and importance attached to the account of the daily activities and home situation of the client. We also found no direct effect from Intention to Behaviour: assessment. This could mean that the questions to measure intentions (Intention) in the questionnaire were insufficiently attuned to behaviour during the assessment interview (Behaviour: assessment). In addition, the absence of direct effects between Attitude and Intention on the one hand and Intention and Behaviour: assessment on the other, may be caused by the narrow distribution of the variable Intention; the majority of insurance physicians scored high on the three additive scales that formed the latent variable Intention [27]. Finally, it is important that the scales and dimensions that are loaded from a specific latent variable (a measured ASE construct) can show positive and negative relationships with the scales and dimensions of another latent variable. As a consequence, relationships between measurements of the ASE constructs may not be found.

The results of the exogenous variables show that insurance physicians who have trained in a medical specialization (besides training as an insurance physician) differ from insurance physicians who have not. Training in another medical specialization turned out to be connected with being more influenced by others (Social norm), higher Self-efficacy and a less stringent, guideline-based, disorder-oriented approach (Behaviour: assessment). These doctors listen to the opinions of other people but their confidence in their own professional ability makes adherence to the rules less important to them.

Insurance physicians who, relatively speaking, carry out more assessments per week seem to pay less heed to the opinions of others. They also have confidence in their professional ability and a less strong professional intention whereby they do assume a stringent, guideline-based, disorder-oriented approach. Steenbeek et al. [27] showed that insurance physicians experience serious barriers amongst which work pressure was most frequently cited. If more working hours per week is indeed related to heavier work pressure, this paper shows that this does not result in lower self-efficacy but rather in a more stringent, guideline-based, disorder-oriented approach.

Steenbeek et al. [27] found that half of the insurance physicians in the study did not have a positive opinion of the Work and Income (labour capacity) Act. This could be related to the years of experience. Our study showed that insurance physicians who have been longer on the job have a less positive attitude to the profession and the system. As noted earlier, Barriers is negatively connected and Knowledge is positively connected with Attitude. This fits in with the result that insurance physicians with more years of experience pay little heed to the opinions of others and demonstrate high self-efficacy, but they also say that there is a shortage of medical data and third-party information in the assessments.

A large quota of clients from the industrial sector is connected with a more stringent, guideline-based, disorder-oriented approach whereas a large quota of clients from the retail sector is connected with a less stringent, guideline-based, disorder-oriented approach. These findings may have something to do with the culture of the organizations where the insurance physicians worked before the Employee Insurance Benefits Agency was founded in 2002. At that time the tasks were performed by Industrial Insurance Associations, which were autonomous organizations with their own sector and culture [53].

### Other studies

Several studies have investigated the relationship between elements of the Theory of Reasoned Action and the Theory of Planned Behaviour (i.e. elements of the ASE model [27,54]) for physicians and healthcare workers in general. These studies were conducted in specific curative settings such as clinical or primary care [55-57] or, in the case of a systematic review study, in a curative healthcare setting in general [58-60]. Most of them found a relationship between Attitude and Intention, between Social norm and Intention, and - if self-reported behaviour was included in the study - between Intention and Behaviour. They conclude that there is a predictable relationship between the intentions of a health care professional and their subsequent behaviour. However, special care should be given to methodological issues, especially to better define the context of behaviour performance of physicians and health care workers.

Our measurements for Intention and Behaviour: assessment reflect to what extend insurance physicians follow the rules. Therefore, studies that show specific relevance to the present study are TPB-based studies on guideline adherence by health care professionals [61-68]. These studies show that social norm (or subjective norm) is often related to intention and seldom to behaviour. In our study we also found a relation between Social norm and Intention, and not between Social norm and Behaviour. In addition, these other studies showed that attitude, perceived behavioural control and self-efficacy are related to both intention and behaviour. In our study, we found that Self-efficacy was related to both Intention and Behaviour: process. However, Attitude was only related to Behaviour: assessment, and not to Intention. Bonetti et al [64] found that higher perceived control and higher self-efficacy in diagnosing and treatment was related to less adherence to the guideline. We found similar results for insurance physicians who have trained in a second specialization. In contrast to our findings Bonetti et al. [64] also found a relation with intention.

We know of only two studies that investigated self-reported behaviour in a non-curative, occupational health setting on the basis of elements of the ASE model. In a study among occupational physicians Rebergen [34] found no relation between self-reported guideline adherence and performance rates. Self-reported guideline adherence correlated significantly with perceived behavioural control, Social norm and positive job stress. In an earlier study of the process of sick-leave assessment among insurance physicians Croon and Langius [33] found relationships between 'Attitudes' and 'Belief in the importance' of certain issues (i.e. Intention) on the one hand and between 'Attitudes' and 'Belief in importance' with 'working styles' (i.e. self-reported preference in behaviour) on the other.

Armitage and Conner [69] found in a meta-analysis of TPB studies that around 27% of the variance in behaviour and 39% of that in intention were explained. In our path model, only 7% of the variance of intention and about 14% to 15% of that of both behaviour variables were explained. This is poor considering the explained variances in the mentioned meta-analysis. Despite the many variables included in our model, most of the variance of intention and behaviour is unaccounted for, i.e. is due to unmeasured influences or/and measurement errors. This raises the question about the validity of the ASE-constructs in the investigated path model. The original 48 additive scales and dimension scores were investigated by Steenbeek et al. [27] and were considered as valid, because of good properties. We used these scales and dimensions scores for the measurements of more general latent ASE-constructs in our model. It is possible that the measurement of the latent ASE-constructs in our model is on such general, high level, that this produced relatively weak relations between these ASE-constructs in our estimated path model.

### Strength and weaknesses

One of the strengths of this study is that the response to the survey was considerably higher than for most other surveys among insurance physicians. Another is that we managed to measure the latent ASE constructs, which were interpretable, and in relating them in a good fitting path model.

One of the weaknesses is the cross-sectional research design, which does not allow for the analysis of causal relationships. Another is that we may not have sufficiently synchronized the questions for the various ASE constructs. For example, the theory on intention and behaviour says that intention is an important predictor of behaviour. We probably did not sufficiently define Intention for our goal: explain the assessment behaviour of insurance physicians (Behaviour: assessment). If we had, Intention might have played a more pronounced role in the model. On the other hand, we did find a relationship between Intention and behaviour related to the assessment process (Behaviour: process). A final weakness relates to the fact that our final model was established on the basis of certain specification choices. It is theoretically possible that other choices would result in another fitted model. We did, however, explore some of the alternative models to arrive at our final model. However, since a lot of modification went on in the model-building process, the findings would need to be replicated, most preferably by confirmatory path analysis in an independent sample. Especially, it is necessary to replicate the scales and dimensions, which we used in the measurement models in this study for the ASE-constructs. Perhaps, items could be developed that distinguish between specific goals and their saliency of intention of social insurance physicians, In addition, items should be developed that describe behaviour more specifically in terms of the Target, Action, Context and Time (TACT) principle [70].

### Practical relevance

In 2004 the Dutch Council for Health Care Research (CHR), a government body, stressed the need to research the variation in disability assessments performed by insurance physicians in the Netherlands [71]. Relevant information could possibly be acquired if the assessments of the Dutch insurance physicians in this study as well as the effects of their work environment and client characteristics could be linked to the outcomes of compensation claims for loss of income due to diminished work capacity. Disability assessment has been the subject of various studies in the Netherlands [24,25,72-74], but these have taken place in a laboratorial rather than a 'real life' setting. If, for example, Attitude (towards both the profession and the social security system) turns out to be strongly related to the outcome of disability assessments, it would be important for social insurance physicians to receive awareness training so that they can handle the potential influence of their opinions when assessing the functional disabilities of a client.

## Conclusion

In conclusion: we found associations in a path model of Attitude and Intention with self-reported Behaviour related to the process and content of occupational disability assessments by insurance physicians. However, the results from this study do not fully confirm the relevance of the Attitude - Social norm - Self-efficacy model in this setting because important associations of the ASE model were not supported. Further research is needed to determine whether the ASE variables measured at the level of insurance physicians are actually associated with the outcome of the occupational disability assessments of clients. If so, we can investigate if assessment behaviour can be influenced in order to limit systematic inter-doctor variation in the outcome of disability assessments.

## Competing interests

The authors declare that they have no competing interests.

## Authors' contributions

AJMS and RS performed the statistical analysis and wrote the manuscript. HM, JRA, HK and JB revised and commented on the manuscript. RS, HM, HK and JB coordinated the data collection. HK provided the logistics for the questionnaire. All authors read and approved the final manuscript.

## Pre-publication history

The pre-publication history for this paper can be accessed here:

http://www.biomedcentral.com/1471-2458/11/576/prepub

## Supplementary Material

Additional file 1 **Scales and dimensions**. Meaning of all measurements (28 scales and 20 dimensions). Click here for file

Additional file 2 **The development of the model**. Research model, adjusted model and final path model. Click here for file
