# Supplementary material for: Can self-reported disability assessment behaviour of insurance physicians be explained? Applying the ASE model
Source: BMC Public Health. 2011 Jul 19;11:576. doi: 10.1186/1471-2458-11-576 (PMC3155499; doi:10.1186/1471-2458-11-576)
Supplement: Additional file 1 — Scales and dimensions. Meaning of all measurements (28 scales and 20 dimensions). [file 1471-2458-11-576-S1.DOC]

# Additional file 1: Scales and dimensions

Validated scales are accompanied by a literature reference: a higher score in these scales means that the respondent scores higher for the subject under investigation.

*Attitude*

Attitude was measured with five additive scales (numbered 1 to 5) and two Homals dimensions (numbered 6 and 7):

1. ‘Job satisfaction’ [29]
2. ‘Positive attitude towards WIA’ (higher score = more positive)
3. ‘Social security system just’ (higher score = stronger agreement)
4. ‘Quality: development of skills important’ (higher score = stronger agreement)
5. ‘Quality: support by management important’ (higher score = stronger agreement)
6. ‘Recovery time: client still has some energy left after work’ (higher score = stronger agreement)
7. ‘Recovery time: good relationship with client’ (higher score = stronger agreement).

*Social Norm*

Social Norm was measured with three additive scales (1 to 3) and two Homals dimensions (4 and 5):

1. 'Opinion of the employer (UWV) and employee representative bodies important’ (higher score = stronger agreement)
2. ‘Colleagues’ opinion important’ (higher score = stronger agreement)
3. ' Society’s opinion important’ (higher score = stronger agreement)
4. ‘Managing by reference to quality rather than quantity’ (higher score = relatively more importance attached to quality-based management than quantity-based management).
5. ‘Managing less by reference to production targets and outcomes’ (higher score = less importance attached to management based on production targets and outcomes).

*Self-efficacy*

Self-efficacy is measured by the ten questions formulated by Scholz et al. [30], modified to take account of the work of insurance physicians. The questions relate specifically to self-efficacy during the disability assessment interview.

*Barriers and stimuli*

Barriers were measured with 9 additive (1-9) scales and 3 Homals dimensions (10-12).

1. Work pressure [31].
2. Emotional workload [32].
3. Decision-making authority [33,34].
4. Emotional exhaustion [35].
5. ‘Office culture: good cooperation’ (higher score = better cooperation) [29].
6. ‘Office culture: sufficient co-determination’ (higher score = better co-determination) [29].
7. ‘Quality: influence of staff physician beneficial’ (higher score = more beneficial).
8. ‘Quality: influence of refresher training and consultation beneficial’ (higher score = more beneficial).
9. ‘Quality: influence of manager beneficial’ (higher score = more beneficial).
10. ‘Quality: influence of legislation and reorganizations not adverse’ (higher score = quality is less adversely affected by legislation and reorganizations).
11. ‘Quality: influence of guidelines not adverse and production target not beneficial’ (higher score = quality is less adversely affected by guidelines and is not positively affected by production targets).
12. ‘Many difficult clients/cases’ (higher score = more clients whom the physician experiences as difficult).

*Knowledge*

Knowledge was measured with one additive scale (1) and three Homals dimensions (2-4):

1. ‘Sufficient information from the occupational physician’ (higher score = the more sufficient the information).
2. ‘Possessing, requesting and using insufficient information’ (higher score = the medical information is not always sufficient, is not always requested from third parties and is not always taken into account in the assessment).
3. ‘Insufficient medical information and knowledge’ (higher score = the less sufficient the medical information and knowledge).
4. ‘Sufficient knowledge, reintegration report less often supplements medical information’ (higher score = the reintegration report supplements the medical information less frequently).

*Intention*

Intention was measured with three additive scales (1-3):

1. ‘Stimulate recovery and return to work’ (higher score = more importance attached to stimulating recovery, return to work, self-perception and reintegration).
2. ‘Basic premises: residual capacity’ (higher score = more importance attached to residual capacity, sickness, disorders, limitations and handicaps).
3. ‘Basic premises: client’s account and home circumstances’ (higher score = more importance attached to a consistent and verified account of daily activities and information about the client’s home circumstances).

*Behaviour: process*

Behaviour reflecting the assessment process was measured with 3 additive scales (1-3) and 5 dimensions (4-8). The additive scale and two dimensions measuring conflict handling are based on Dreu et al. [36].

1. Dedication [37].
2. ‘Technical interview: describe object and procedure’ (higher score = greater emphasis at the beginning of the interview on the purpose and procedural aspects of the interview).
3. ‘Conflict handling: seek compromise’ (higher score = compromise sought more frequently with client).
4. ‘Interview management: client decisive’ (higher score = the client determines the order of events more often than the insurance physician).
5. ‘Interview: limitations not checked’ (higher score = the physician checks the client’s limitations less frequently).
6. ‘Interview: respond to client’ (higher score = more frequent response to subjects raised by the client).
7. ‘Conflict handling: engage in confrontation’ (higher score = more likely to engage in confrontation).
8. ‘Conflict handling: play down differences’ (higher score = tries more often to circumvent differences of opinion and play down their importance).

*Behaviour: assessment*

Behaviour reflecting the assessment was measured with three additive scales (1-3) and five dimensions (4-8).

1. ‘Comply with permanent full disability rules’ (higher score = more frequent compliance).
2. ‘FAL (Functional ability list): takes account of client’ (higher score = focuses more often on the complaints raised by the client, what the client can really do, the client’s difficult home circumstances and limitations experienced by the client).
3. ‘FAL: consults with labour expert when not necessary’ (higher score = consults more often with labour expert in circumstances where the client is unable to work or does not belong in the benefits category).
4. ‘FAL and recovery time: stringent/formalistic approach’ (higher score = adopts more often a formalistic and stringent approach to drawing up the FAL and takes no account of the client or his recovery time).
5. ‘FAL and recovery time: focus on impairments’ (higher score = pays more attention when drawing up the FAL to limitations caused by disorders, particularly in the light of consistency, and takes no account of a possible deterioration in the client’s health).
6. ‘Client approach: involved with and time for’ (higher score = makes more time for and is involved with the client).
7. ‘Client approach: time for account of daily activities and reporting’ (higher score = the client is questioned in detail more often about his daily activities and more time is taken for reports).
8. ‘Client approach: too little time, but involved with’ (higher score = more likely to have too little time to draw up a proper report and to question the client about his daily activities, but feels involved with the client).
