# Supplementary material for: Can self-reported disability assessment behaviour of insurance physicians be explained? Applying the ASE model
Source: BMC Public Health. 2011 Jul 19;11:576. doi: 10.1186/1471-2458-11-576 (PMC3155499; doi:10.1186/1471-2458-11-576)
Supplement: Additional file 2 — The development of the model. Research model, adjusted model and final path model. [file 1471-2458-11-576-S2.DOC]

# Additional file 2: The development of the model

We began by testing our research model according to the ASE theory (see Figure 1). The following relationships were modelled between endogenous variables:

Attitude 🡪 Intention

Social norm 🡪 Intention

Self-efficacy 🡪 Knowledge, Intention

Knowledge 🡪 Intention, Behaviour Process, Behaviour Assessment

Barriers 🡪 Intention, Behaviour Process, Behaviour Assessment

Intention 🡪 Behaviour Process, Behaviour Assessment

Behaviour P 🡪 Behaviour Assessment

The model fit was poor (Chi-square=85.8, df=45, p=0.000, RSMEA=0.063, CFI=0.800, SRMR =0.067).

We then adjusted the model. Non-significant parameters were removed and new parameters were added if justified on the basis of theoretical insight and modification indices. The following relationships were modelled between endogenous variables:

Attitude 🡪 Behaviour Process, Behaviour Assessment

Social norm 🡪 Intention,

Self-efficacy 🡪 Intention, Behaviour Process, Behaviour Assessment

Knowledge 🡪 Behaviour Assessment

Barriers 🡪 Behaviour Process

Intention 🡪 Behaviour Process

Behaviour P 🡪 Behaviour Assessment

This adjusted model still showed a fit, which – particularly in view of the size and the distribution of the residuals – could be further improved. An important adjustment was still needed to arrive at an acceptable final model. The modification index of 8.03 pointed to a significant effect by Behaviour Assessment on Self-efficacy. In a comparison of alternative models Lisrel clearly showed that a model in which Self-efficacy had a direct effect on Behaviour Assessment had a significant worse fit (Chi-square=42.5, df=48, p=0.697, RSMEA=0.000, CFI=1.00, SRMR=0.046) than the model with a direct effect from Behaviour Assessment to Self-efficacy (Chi-square = 37.5, df =48, p=0.863, RSMEA=0.000, CFI=1.00, SRMR=0.041). Furthermore, another model in which Self-efficacy had a direct effect on Behaviour Assessment and vice versa, a very high correlation between the concerning parameter estimates resulted (0.91), leading to unreliable results of the parameter estimates. The model with a direct effect of Behaviour Assessment on Self-efficacy only was therefore chosen as the final model.
